# Bone mineral: new insights into its chemical composition

**DOI:** 10.1038/s41598-019-44620-6

**Published:** 2019-06-11

**Authors:** Stanislas Von Euw, Yan Wang, Guillaume Laurent, Christophe Drouet, Florence Babonneau, Nadine Nassif, Thierry Azaïs

**Affiliations:** 1 Sorbonne Université, CNRS, Collège de France, Laboratoire de Chimie de la Matière Condensée de Paris (LCMCP), 4, place Jussieu, F-75005 Paris, France; 2 Present Address: Trinity College Dublin, Trinity Centre for Bioengineering (TCBE), Trinity Biomedical Sciences Institute, 152-160 Pearse Street, Dublin 2, Ireland; 3 CIRIMAT, Université de Toulouse, CNRS, INP-Ensiacet, 4 allée Emile Monso, F-31030 Toulouse, France

**Keywords:** Biomineralization, Solid-state NMR

## Abstract

Some compositional and structural features of mature bone mineral particles remain unclear. They have been described as calcium-deficient and hydroxyl-deficient carbonated hydroxyapatite particles in which a fraction of the PO_4_^3−^ lattice sites are occupied by HPO_4_^2−^ ions. The time has come to revise this description since it has now been proven that the surface of mature bone mineral particles is not in the form of hydroxyapatite but rather in the form of hydrated amorphous calcium phosphate. Using a combination of dedicated solid-state nuclear magnetic resonance techniques, the hydrogen-bearing species present in bone mineral and especially the HPO_4_^2−^ ions were closely scrutinized. We show that these HPO_4_^2−^ ions are concentrated at the surface of bone mineral particles in the so-called amorphous surface layer whose thickness was estimated here to be about 0.8 nm for a 4-nm thick particle. We also show that their molar proportion is much higher than previously estimated since they stand for about half of the overall amount of inorganic phosphate ions that compose bone mineral. As such, the mineral-mineral and mineral-biomolecule interfaces in bone tissue must be driven by metastable hydrated amorphous environments rich in HPO_4_^2−^ ions rather than by stable crystalline environments of hydroxyapatite structure.

## Introduction

Bone is a natural composite material whose main components are mineral and organic matrices^1,2^. The mature mineral matrix is in the form of nanosized, elongated platelet-like carbonated calcium phosphate particles whose elongated direction is preferentially aligned along the long axis of type I collagen fibrils^3^. Those collagen fibrils compose the organic matrix, together with different types of biomacromolecules, including proteoglycans and non-collagenous proteins (NCPs)^4–6^. Bone mineral is known to possess compositional and structural similarities with synthetic stoichiometric hydroxyapatite (HA) mineral, Ca_10_(PO_4_)_6_(OH)_2_. However, in contrast to stoichiometric HA, bone mineral is structurally disordered, and compositionally nonstoichiometric due to the presence of a substantial amount of anionic (*e*.*g*., HPO_4_^2−^, CO_3_^2−^, Cl^−^) and cationic (*e*.*g*., Na^+^, Mg^2+^) species, together with the presence of ion vacancies into the crystal lattice. For instance, CO_3_^2−^ ions, whose weight proportion can reach up to ∼5–9% in bone mineral, can occupy the PO_4_^3−^ (called B-type substitution - major) and/or OH^−^ (A-type substitution - minor) sites within the hydroxyapatite’s crystal lattice. In addition, it was reported that the PO_4_^3−^ lattice sites may also be occupied by a fraction of monohydrogen-phosphate (HPO_4_^2−^) ions. As a consequence, it is generally accepted that “the deficit in negative charge caused by the replacement of PO_4_^3−^ by either CO_3_^2−^ or HPO_4_^2−^ can be compensated by the loss of positive charge, as through removal of Ca^2+^ from the lattice”^7^. Furthermore, beside their presence within the hydroxyapatite’s crystal lattice, HPO_4_^2−^ ions were also proposed to be present in the so-called amorphous surface layer that coats both biological^8–11^ (bone, dentin) and biomimetic^11–16^ nanocrystalline hydroxyapatite particles. Lastly, HPO_4_^2−^ ions in bone could also originate from the presence of octacalcium phosphate (OCP), Ca_8_(HPO_4_)_2_(PO_4_)_4_·5H_2_O and OCP-like domains. Indeed, OCP was identified as a possible transient precursor phase of bone hydroxyapatite^17–19^, whilst OCP-like domains were also proposed as a component of bone mineral^20–22^.

The presence of HPO_4_^2−^ ions was initially proposed and quantified in synthetic hydroxyapatites based on spectroscopic analysis^23^, together with bulk chemical analysis of pyrolyzed samples^24^ for which the pyrophosphate ions created by the condensation of two HPO_4_^2−^ ions after dehydration upon heating were titrated. The latter method was also used to quantify HPO_4_^2−^ ions incorporated into bone mineral^25,26^. A combination of chemical and spectroscopic analysis of bone mineral samples of different age and origin (rat, calf and cow) was further undertaken. It lead to the average chemical formula^27^ (neglecting the most minor substitutions) proposed by Legros *et al*. in 1987 for mature cortical bone mineral which is still considered as a reference formula to this day: Ca_8.3□1,7_(PO_4_)_4.3_(HPO_4_ or CO_3_)_1.7_(OH or ½ CO_3_)_0,3□1.7_. Since then, the presence of HPO_4_^2−^ ions in bone mineral was also proposed based on vibrational spectroscopic analyses^25–27^. Indeed, thanks to the comparison with hydrogen-phosphate-containing calcium phosphate mineral standards such as brushite (Dicalcium Phosphate Dihydrate, DCPD; CaHPO_4_.2H_2_O) and octacalcium phosphate, Fourier Transform-Infrared (FT-IR) analyses of bone (chicken) and enamel (pig) have revealed that biological hydroxyapatites exhibit characteristic adsorption bands that were attributed to HPO_4_^2−^ions^28,29^. Solid-state Nuclear Magnetic Resonance (ssNMR) spectroscopy has also been used to study bone mineral and, in particular, its chemical structure^30,31^, its hydrophilicity^32^, and its interaction with bioorganic molecules^33–37^. The ^31^P NMR chemical environments have been probed; and early studies on bone tissue samples have also suggested the presence of HPO_4_^2−^ ions in bone mineral based on the measurement of chemical shift anisotropy (CSA) parameters^38^: they were found to differ from those of apatitic PO_4_^3−^ ions but to be similar with those found for brushite. Similarly, ^1^H-^31^P dipolar-based ssNMR experiments^39,40^ revealed two different behaviours in various bone tissue samples (chicken, bovine and rabbit) attributed to HPO_4_^2−^ *vs* PO_4_^3−^. Lastly, the presence of HPO_4_^2−^ ions in bone mineral was also proposed based on ^1^H NMR chemical shift considerations: two-dimensional (2D) {^1^H}^31^P heteronuclear correlation (HetCor) NMR experiments were performed to indirectly detect the ^1^H NMR chemical environments of bone^41–43^ and dentine^9^ mineral that were then compared with the ^1^H NMR chemical environments of brushite, monetite (Dicalcium Phosphate Anhydrous, DCPA; CaHPO_4_) and octacalcium phosphate^44^.

Many efforts have therefore been made over the years to identify, localize and quantify HPO_4_^2−^ ions within bone mineral. While their presence in bone mineral is now accepted, both their localisation and quantification are still being debated^45^. This prevents the design of an accurate chemical and structural model of mature bone mineral particles which will not only provide design principles for the next generation of alloplasts for bone regeneration, but will also facilitate the understanding of bone mineral chemistry and reactivity *in vivo*. Hence, the present study aims to more precisely assess the identification, the localisation, and the quantification of HPO_4_^2−^ ions in bone mineral. To this end, we used a combination of dedicated ssNMR techniques from intact mature bone tissue samples together with synthetic reference samples. In particular, the {^1^H-^31^P}^1^H double cross polarization (CP) ssNMR experiment was used to suppress the proton signal from the bone organic matrix, and, therefore, to selectively record the ^1^H NMR spectrum of bone mineral. The variation of the ^31^P → ^1^H contact time in the {^1^H-^31^P}^1^H double CP experiment, followed by numerical modelling and calculations allowed us to determine the H•••P distances within the HPO_4_^2−^ ions identified in bone mineral. These distances were subsequently compared with those determined for known inorganic POH groups found in HPO_4_^2−^ ions from monetite. Further ssNMR investigations including ^1^H-^1^H double quantum-Single quantum experiments have also been undertaken to study the localization of the HPO_4_^2−^ ions present in a bone mineral proxy sample. Lastly, a single pulse ^31^P ssNMR spectrum of bone mineral recorded under quantitative conditions was recorded to quantify these HPO_4_^2−^ ions with respects to the overall amount of inorganic phosphate ions that compose bone mineral.

## Methods

### Samples preparation

Cortical bone tissue samples were harvested from healthy 2-year-old sheep; and were extracted from the distal femoral metaphysis. The animal experiments were approved by the IMMR’s Institutional Animal Care and Use committee (IACUC) and performed in accordance with relevant guidelines and regulations. The IMMR received an agreement (n° 75-14-01) on September 08th, 2008 for period of 5 years by the “Sous-Direction de la protection Sanitaire” of the French Authorities. Fresh bone tissue samples were analysed within two hours following their extraction from the animal. This delay corresponds to the trip from the hospital where the bone tissue samples were harvested to our lab. In the meantime, the bone samples were conserved intact in a sealed vial at ambient temperature. The dry bone tissue samples were obtained once the fresh bone tissue samples were dehydrated at ambient temperature in a laminar flow hood for one night.

Monetite (DCPA) was obtained by mixing calcium carbonate (CaCO_3_) and an aqueous phosphoric acid solution (H_3_PO_4,_ 85 wt. %) in water (ratio Ca/P = 1). The mixture was placed in an autoclave at 150 °C for 48 h. The resulting precipitate was then washed with ethanol and dried at 37 °C.

OctaCalcium Phosphate (OCP) was prepared according to the protocol of Bigi *et al*. (2004)^46^. Briefly, 500 mL of an aqueous solution containing 40 mM Ca(CH_3_COO)_2_ was added dropwise to 1500 mL of an aqueous solution containing 6.6 mM of Na_2_HPO_4_ and 6.6 mM of NaH_2_PO_4_ with a starting pH of 5. The reaction was performed at 70 °C, and no stirring was applied. Fifteen minutes after the end of the addition, the precipitate was centrifuged, washed three times with deionized water, and then dried at 37 °C

A biomimetic Carbonated HydroxyApatite (CHA-SBF) sample was precipitated directly from a modified simulated body fluid solution (SBF) inspired from human blood plasma. A solution 1.5 times more concentrated compared to standard SBF was prepared (1.5 × SBF)^47^. Briefly, 1 L of this solution was frozen at −20 °C for 1 night. Then, the solution was thawed and conserved at 5 °C for 1 month. The resulting nanoparticles were recovered by centrifugation and finally dried at 37 °C. To study CHA-SBF in wet conditions, about 10 µL of deionized water has been directly added to the rotor prior to NMR analysis.

A well-crystallized hydroxyapatite (HA) sample which, unlike CHA-SBF, is exempt of amorphous surface layer, but which is composed of HPO_4_^2−^ ions that only occupy the PO_4_^3−^ sites within the hydroxyapatite’s crystal lattice was also prepared. This sample, named here HPO_4_^2^^−^-substituted HA, corresponds to so-called apatitic tricalcium phosphate, with the following chemical formula: Ca_9_(PO_4_)_5_(HPO_4_)(OH). It thus contains 1/6^th^ (~ 17%) of protonated HPO_4_^2−^ ions out of the total phosphate content^48^. This sample was obtained by hydrothermal treatment by converting β−tricalcium phosphate Ca_3_(PO_4_)_2_, of particle size <125 µm, with water vapor at 160 °C for 3 days in an autoclave.

### FT-IR analyses

Fourier Transform-Infrared (FT-IR) spectra were recorded at room temperature using a Nicolet Magna FT-IR spectrometer in ATR mode, in the range of 650–4000 cm^−1^ and at a resolution of 4 cm^−1^. The weight proportion of CO_3_^2−^ ions in bone mineral from our 2-year-old sheep bone tissue sample was evaluated according to the FT-IR analysis procedure described by Grunenwald *et al*.^49^.

### Solid-state NMR

Solid-state Nuclear Magnetic Resonance (ssNMR) experiments were performed on an Avance 300 Bruker spectrometer (7.0 T) using a 4 mm double resonance magic angle spinning (MAS) probe head. 3-4mm-thick pieces of cortical bone samples were packed into a 4 mm (O.D.) NMR zirconia rotor and spun at a Magic Angle Spinning (MAS) frequency ν_MAS_ = 14 kHz. The temperature in the NMR probe was kept at 25 °C during all analysis periods. The recycle delays in the one-dimentional (1D) {^1^H}^31^P cross polarization (CP), two-dimentional (2D) {^1^H}^31^P Heteronuclear Correlation (HetCor), 1D {^1^H-^31^P}^1^H double CP; and in the 2D ^1^H-^1^H double quantum-Single quantum experiments, were set to 2 sec (bone and CHA-SBF), 10 sec (monetite) and 30 sec (HPO_4_^2^^−^-substituted HA). The 2D {^1^H}^31^P HetCor experiments were recorded with a contact time of 1000 µs and 80 scans for each 100 t_1_ increments. The radio frequency (RF) field (B_1_) applied during the CP steps was ν_RF_(^1^H) = 70 kHz and ν_RF_(^31^P) = 50 kHz. The {^1^H-^31^P}^1^H double CP experiments were recorded with identical RF fields. The sequence consists of two consecutive CP transfers, and is schematically described in Fig. S1. Following the first transfer (during t_CP_1), the ^31^P magnetization is flipped back to the z direction through a 90° pulse. The ^1^H residual signal is eliminated by two low power pulses phase shifted by 90° at ν_RF_ = ν_MAS_/2 = 7 kHz (HORROR condition^50^). The length of each pulse corresponds to the length of the ^1^H free induction decay (∼ 10 ms). This saturation step not only enables suppression of all unwanted ^1^H signals; but it also, within the model of thermal reservoirs^51^, enables transformation of the proton bath into a hot reservoir into which the ^31^P magnetization can be back-transferred from the cold reservoir. After this step, the ^31^P magnetization is then flipped back into the transverse plane through a 90° pulse and a second CP transfer is then applied (during t_CP_2) prior to ^1^H acquisition. In regard to the CP dynamics experiments, the Hartmann-Hahn condition of the first CP ^1^H → ^31^P transfer was set through a tangential ramp on the ^1^H channel in order to maximize the ^31^P signal^52^. The Hartmann-Hahn condition of the second CP ^31^P → ^1^H transfer was set through square pulses on both channels. The ^1^H and ^31^P radio frequency field strengths were matched to the first spinning sidebands (n = ±1) of the Hartmann-Hahn matching profile (Fig. S2). High power ^1^H decoupling was applied during acquisition (60 kHz of RF field strength, spinal 64). Regarding the NMR signal processing, no line broadening (LB) was employed to process the {^1^H-^31^P}^1^H double CP free induction decay (FID); while a line broadening of 30 and 100 Hz was employed for the 2D {^1^H}^31^P HetCor experiments in the F2 and F1 dimensions, respectively. Two dimensional ^1^H-^1^H double quantum-Single quantum NMR spectra were recorded using the Back-to-Back (BABA) excitation scheme based on the recoupling of ^1^H homonuclear dipolar couplings^53^. The ^1^H RF field was 70 kHz. The recoupling delay was synchronized to the rotor rotation period (71.4 µs). ^1^H chemical shifts were referenced to TetraMethylSilane (TMS) at δ^1^H = 0.0 ppm, whereas ^31^P chemical shifts were referenced to H_3_PO_4_ (85% w/w aqueous solution) at δ^31^P = 0.0 ppm.

## Results and Discussion

### Identification of HPO_4_^2−^ ions in bone mineral

**Figure 1:**
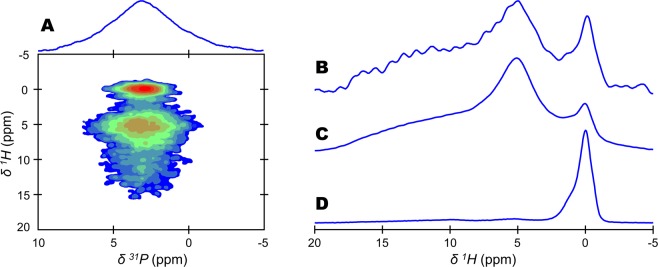
Detection of hydrogen-bearing species in bone mineral. ^1^H-^31^P cross polarization (CP) based magic angle spinning (MAS) solid-state Nuclear Magnetic Resonance (ssNMR) spectra of a dry 2-year-old sheep bone tissue sample. (**A**) two-dimensional (2D) {^1^H}^31^P Heteronuclear Correlation (HetCor) spectrum (contact time, t_CP_ = 1000 µs). Signal intensity increases from blue to red. (**B**) ^1^H projection of the vertical (F1) dimension of the 2D {^1^H}^31^P HetCor spectrum shown in (**A**). {^1^H-^31^P}^1^H double CP MAS spectra recorded with the following contact times: (**C**) t_CP_1 = t_CP_2 = 1000 µs; and, (**D**) t_CP_1 = t_CP_2 = 15000 µs. The total experimental time was the same in each experiment (i.e., 9 hours).

The direct solid-state Nuclear Magnetic Resonance (ssNMR) detection of the protons localized in bone mineral from an intact bone tissue sample is not possible. This is due to the presence of the extracellular organic matrix whose different signals dominate the ^1^H single pulse (SP) ssNMR spectrum of a bone tissue sample^54^. However, the possibility to reveal atomic-scale spatial proximities among hydrogen and phosphorus nuclei in the two-dimensional (2D) {^1^H}^31^P Heteronuclear Correlation (HetCor) ssNMR experiment allows for probing bone mineral hydrogen environments through the analysis of the F1 dimension (Fig. 1A). Unfortunately, this experiment is time consuming and gives rise to a ^1^H projection of the vertical (F1) dimension with a relatively poor signal-to-noise ratio (S/N) and a low digital resolution (Fig. 1B). To overcome these limitations, we used the one-dimensional (1D) {^1^H-^31^P}^1^H double cross polarization (CP) ssNMR experiment. It consists of a double CP transfer conducted in a “there-and-back” manner (^1^H→^31^P→^1^H) (Fig. S1). First, this experiment allowed us to obtain ^31^P-filtered ^1^H ssNMR spectra of bone mineral from an intact, cortical 2-year-old sheep bone tissue sample with an excellent S/N despite a relatively short acquisition time (i.e., 9 hours) (Fig. 1C,D). The different ^1^H chemical environments from bone mineral are now readily observable and can be safely analyzed with precision. With regard to the internal crystalline core of bone mineral particles, the hydroxyl ions present in the hydroxyapatite’s crystal lattice are observed in the form of a complex resonance centred at δ(^1^H) = 0.0 ppm. Regarding their amorphous surface layer, structural water molecules and acidic phosphate species present in non-apatitic environments are observable in the form of a single resonance centred at δ(^1^H) = 5.2 ppm and a broad resonance ranging from δ(^1^H) = 7 to 17 ppm^32^, respectively.

**Figure 2:**
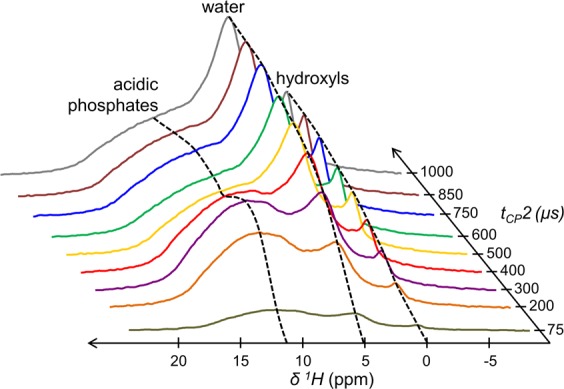
Cross-polarization dynamics of the ^1^H resonances from bone mineral. {^1^H-^31^P}^1^H double cross polarization (CP) magic angle spinning (MAS) solid-state Nuclear Magnetic Resonance (ssNMR) dynamics of a dry 2-year-old sheep bone tissue sample. Contact time 1 (t_CP_1) was fixed at 1000 µs, while contact time 2 (t_CP_2) was varied from 75 µs to 1000 µs. Black dashed lines are guidelines for the eyes.

**Figure 3:**
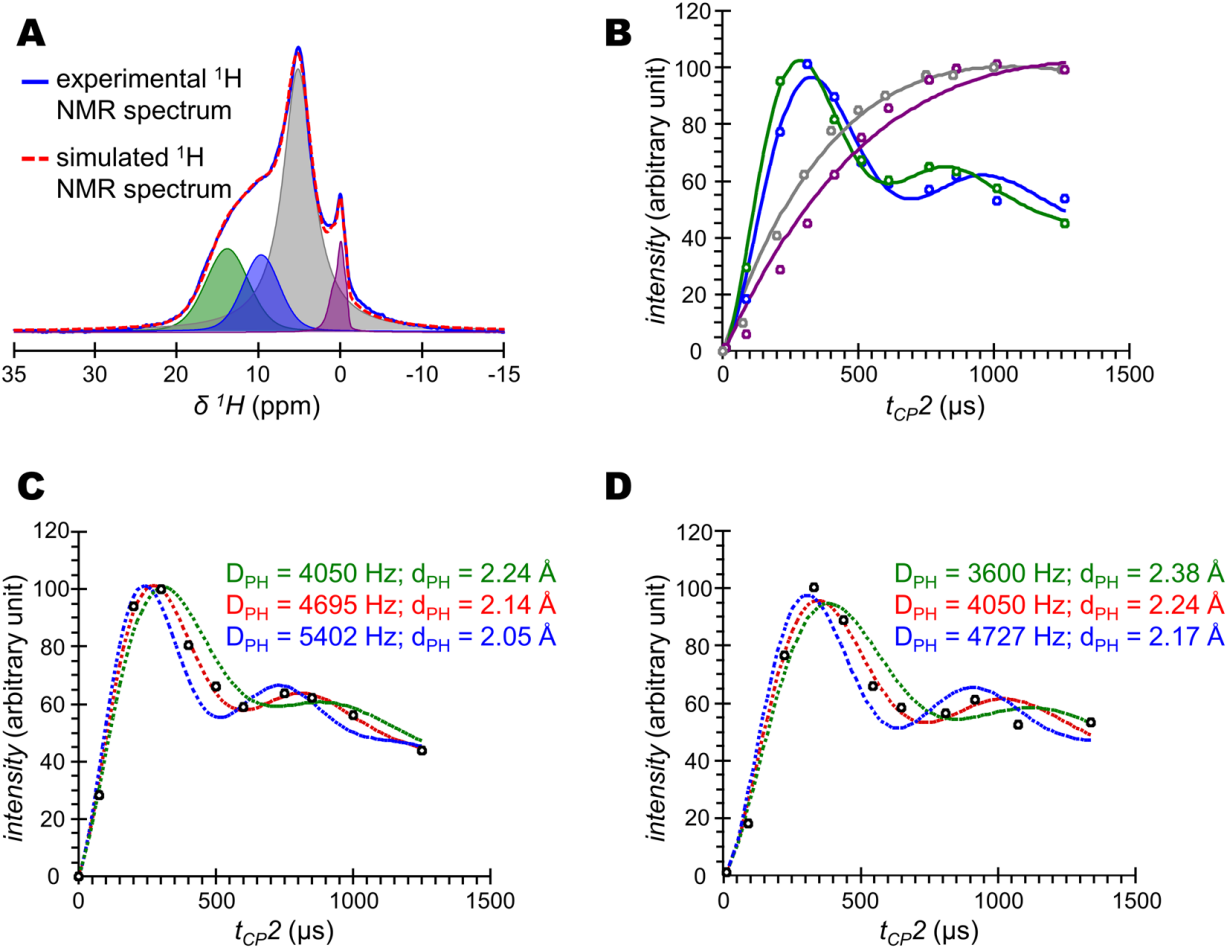
Determination of ^31^P-^1^H internuclear distances within the acidic phosphate species present in bone mineral. (**A**) {^1^H-^31^P}^1^H double cross polarization (CP) magic angle spinning (MAS) solid-state Nuclear Magnetic Resonance (ssNMR) spectrum of a dry 2-year-old sheep bone tissue sample (blue line) and its corresponding fitting (red dashed line). Contact times 1 (t_CP_1) was 1000 µs, while contact time 2 (t_CP_2) was 500 µs. (**B**) Numerical modelling of the evolution of the magnetization of the δ(^1^H) = 0 (purple, hydroxyl ions); 5.2 (grey, structural water molecules); 9.8 (blue, acidic phosphate species); and, 14.0 (green, acidic phosphate species) ppm peaks shown in (A). The acidic phosphate species peaks were simulated according to Eq. (1), while the hydroxyl ions and structural water molecules resonances were simulated according to Eq. (3). Numerical modelling according to Eq. (1) of the magnetization evolution of the δ(^1^H) = 14.0 (**C**) and 9.8 (**D**) ppm peaks attributed to acidic phosphate species; together with the calculations of their respective dipolar constant (D_PH_) and P•••H distance (d_PH_).

Second, this experiment allows the investigation of the ^1^H-detected CP dynamics to selectively reveal the nature of the ^1^H nuclei nearby ^31^P nuclei. To this end, the contact time 1 (t_CP_1) was kept fixed at 1000 µs, while the contact time 2 (t_CP_2) was varied from 75 µs up to 1000 µs (Fig. 2). A uniform increase of the magnetization is observed for both the resonances centred at δ(^1^H) = 0.0 and δ(^1^H) = 5.2 ppm (see the black dashed lines), previously attributed to OH^−^ ions and structural H_2_O molecules according to their respective ^1^H NMR chemical shift. In contrast, the evolution of the broad signal in the range of δ(^1^H) = 7–17 ppm initially shows a rapid increase of its magnetization (up to t_CP_2 = 300 µs) and is followed by the presence of an oscillatory behaviour (up to t_CP_2 = 1250 µs - see the black dashed line). This oscillatory behaviour is characteristic of ^1^H-^31^P dipolar (D_P-H_) oscillations^55,56^. The fitting of the corresponding {^1^H-^31^P}^1^H ssNMR spectra at various t_CP_2 is not straightforward due to the overlapping of various resonances. Whereas synthetic HA samples usually exhibit a symmetric OH^-^ resonance^32^; we show here that the OH^−^ resonance of bone mineral is particularly complex and can be fitted as follows: a main peak at δ(^1^H) = 0.0 ppm surrounded by two shoulders peaks at δ(^1^H) = −0.7 and 0.9 ppm (Fig. S3). The residual structural water resonance can be properly fitted with a single peak centred at δ(^1^H) = 5.2 ppm (Fig. S4). In contrast, the broad signal from the acidic phosphate species observable in the range of δ(^1^H) = 7–17 ppm cannot be satisfactorily fitted with a single peak with fixed position and line width, especially at short contact times (see the best fitting results for the various t_CP_2 values in Fig. S4 – left column). However, the fitting results are accurate when two different peaks are used with fixed positions [at δ(^1^H) = 9.8 ± 0.1 and 14.0 ± 0.1 ppm] and fixed line widths (6.2 ± 0.1 and 5.0 ± 0.1 ppm, respectively) (Fig. S4 – right column). We cannot claim that this broad signal is only composed of two peaks corresponding to two distinct proton environments, but it is probably composed of a wide distribution of chemical environments leading to a distribution of NMR chemical shifts. Accordingly, Fig. 3A shows the four peaks that were used to analyse the ^31^P-filtered ^1^H ssNMR spectra of bone mineral for each t_CP_2 values: (*i*) a composite peak centred at δ(^1^H) = 0.0 (purple) and a single peak centred at δ(^1^H) = 5.2 (grey) ppm both characterized by a relatively slow and uniform growth of their magnetizations; and (*ii*) two peaks at δ(^1^H) = 9.8 (blue) and 14.0 (green) ppm both displaying dipolar oscillations (Fig. 3B). In regard to these latter two peaks, the precise match of the Hartmann-Hahn (H-H) profile (Fig. S2) enables numerical modelling of the CP build-up curves according to the single spin pair model accounting for dipolar oscillations resulting from coherent polarization transfer and the impact of ^1^H spin diffusion^57^:

1 $${\rm{M}}({\rm{t}})={{\rm{M}}}_{0}.\exp (-\,{{\rm{t}}}_{{\rm{CP}}}/{{\rm{T}}}_{1{\rm{\rho }}}({}^{{\rm{1}}}{\rm{H}})).[(1-1/2.\exp (-{{\rm{t}}}_{{\rm{CP}}}/{{\rm{T}}}_{{\rm{sd}}})-1/2.\exp (-3{{\rm{t}}}_{{\rm{CP}}}/2{{\rm{T}}}_{{\rm{sd}}}).\,\cos ({{\rm{\pi }}D}_{{\rm{PH}}}\mbox{'}.{{\rm{t}}}_{{\rm{CP}}}))$$

in which M_0_, t_CP_, T_sd_, T_1ρ_(^1^H) and D_PH_’ are the CP intensity, the contact time, the ^1^H spin-diffusion rate constant, the spin-lattice relaxation time in the rotating frame, and the apparent ^1^H-^31^P dipolar coupling, respectively. The internuclear distance between ^1^H and ^31^P nuclei is then readily extracted from D_PH_:

2 $${{\rm{D}}}_{{\rm{PH}}}=({\mu }_{0}.{\rm{h}}.{{\rm{\gamma }}}_{1{\rm{H}}}.{{\rm{\gamma }}}_{31{\rm{P}}})/(16{{\rm{\pi }}}^{3}.{{{\rm{d}}}_{{\rm{PH}}}}^{3})$$

in which D_PH_ = D_PH_’ × √2, d_PH_ is the internuclear distance between the two spins, γ_1H_ and γ_31P_ are the gyromagnetic ratios of the coupled spins, *µ*_0_ is the vacuum permeability, and h is the Planck constant. A dipolar constant (D_PH_) of 4050 Hz was found for the peak centred at δ(^1^H) = 9.8, while D_PH_ = 4695 Hz for the peak centred at δ(^1^H) = 14.0 ppm. In addition, the corresponding H•••P internuclear distances were calculated: 2.24 Å for the former peak and 2.14 Å for the latter peak (Fig. 3C,D, and Table 1). The estimated precision is ± 490 Hz for D_PH_ and ± 0.07 Å for d_PH_. Lastly, in regard to the evolutions of the magnetization of the resonances at δ(^1^H) = 0.0 ppm (OH^-^) and δ(^1^H) = 5.2 ppm (H_2_O); they are readily fitted within the classical I-S model. This is an analytical extension of Eq. 1 for an extended spin system in which the behaviour of the magnetization is dominated by an incoherent transfer from ^31^P to ^1^H as follows^57^:

3 $${\rm{M}}({\rm{t}})={{\rm{M}}}_{0}\cdot (1/(1-{{\rm{T}}}_{{\rm{H}}{\rm{P}}}/{{\rm{T}}}_{1\rho }({}^{1}{\rm{H}})))\cdot (\exp (\,-\,{{\rm{t}}}_{{\rm{C}}{\rm{P}}}/{{\rm{T}}}_{1\rho })-\exp (\,-\,{{\rm{t}}}_{{\rm{C}}{\rm{P}}}/{{\rm{T}}}_{{\rm{H}}{\rm{P}}}))$$

in which M_0_, t_CP_, T_HP_, T_1ρ_(^1^H) are the CP intensity, the contact time, the CP rate constant and the spin-lattice relaxation time in the rotating frame, respectively. A T_HP_ value of 795 µs was found for the OH^-^ resonance, while a T_HP_ value of 522 µs was found for the H_2_O resonance (Table 1).

**Table 1:** Nuclear Magnetic Resonance (NMR) parameters associated with the different proton (^1^H) species detected in the various samples (bone mineral, CHA-SBF and Monetite): ^1^H chemical shifts (δ^1^H); dipolar coupling constants for the ^31^P-^1^H interaction (D_PH_); P•••H internuclear distances (d_PH_); cross-polarization (CP) rate constants (T_HP_); spin-diffusion rate constants (T_df_); spin-lattice relaxation times in the rotating frame (T_1ρ_^1^H). ^a^was calculated according to Equation 3; whereas ^b^was calculated according to Equation 1.

Sample	^1^H species	δ(^1^H) (ppm)	D_PH_ (Hz)	d_PH_ (Å)	T_HP_^a^ (µs)	T_df_^b^ (µs)	T_1ρ_ (^1^H) (µs).
Bone mineral	OH^−^	0.0	—	—	795	—	∞
	H_2_O	5.2	—	—	522	—	∞
	HPO_4_^2−^	9.8	4050 ± 500	2.24 ± 0.07	—	788	1485
	HPO_4_^2−^	14.0	4695 ± 500	2.14 ± 0.07	—	624	1224
CHA-SBF	HPO_4_^2−^	7.0–17.0	4960 ± 500	2.10 ± 0.07	—	665	3531
Monetite	HPO_4_^2−^	13.0	5275 ± 500	2.10 ± 0.07	—	626	∞
	HPO_4_^2−^	15.8	6863 ± 900	1.92 ± 0.14	—	691	∞

The same ssNMR-based approach was also applied to monetite (CaHPO_4_) as a reference sample which is a hydrogen-phosphate-containing calcium phosphate mineral standard (Figs S5 and S7). Dipolar oscillations are also observed for which our numerical modelling and calculations reveal H•••P internuclear distances of 1.92 ± 0.07 and 2.10 ± 0.07 Å within the two inequivalent HPO_4_^2−^ groups present in the monetite’s crystal lattice. Such distances are close to those found for the acidic phosphate species within our 2-year-old sheep bone tissue sample, and are in agreement with the distances measured in monetite^58^ and brushite^59^ through neutron diffraction analysis (i.e., d_PH_ ranging from 2.08 to 2.25 in the HPO_4_^−^ ions of monetite; and d_PH_ = 2.19 Å in the HPO_4_^2−^ ions of brushite). Since previous spectroscopic analyses have shown that the presence of H_2_PO_4_^−^ ions in carbonated hydroxyapatite was unlikely^12^, we can now safely attribute the broad signal in the range of δ(^1^H) = 7–17 ppm observable in bone mineral solely to HPO_4_^2−^ ions.

In addition, the same ssNMR-based approach was also applied to a biomimetic Carbonated HydroxyApatite (CHA-SBF) sample that was precipitated from a modified simulated body fluid solution (SBF) devoid of organic additives. This synthetic bone-like mineral sample displays a similar {^1^H-^31^P}^1^H double CP ssNMR spectrum than bone mineral: a resonance centred at δ(^1^H) = 0.0 ppm attributed to hydroxyl ions; a resonance centred at δ(^1^H) = 5.2 ppm attributed to structural water molecules; and last, a broad signal in the range of δ(^1^H) = 7–17 ppm attributed to acidic phosphate species (Fig. S7). The variation of the second contact time (t_CP_2) also leads to the observation of dipolar oscillations in the acidic phosphate species region. Our corresponding numerical modelling and calculations reveal a dipolar constant (D_PH_) of 4960 ± 500 Hz, and a distance (d_PH_) of 2.10 ± 0.07 Å. These values are similar to those calculated for the acidic phosphate species found in bone mineral (Fig. 3 and Table 1). These results demonstrate that the acidic phosphate species detected in bone mineral from our 2-year-old sheep bone tissue sample are purely inorganic (HPO_4_^−^ ions) and do not originate from POH groups present in bioorganic molecules such as phosphorylated NCPs and phospholipids.

### Localisation of the HPO_4_^2−^ ions in bone mineral

The exact localisation of the structural water molecules and the HPO_4_^2−^ ions within bone mineral particles is still unclear. To date, they are suggested to be present both in the hydroxyapatite’s crystal lattice (internal crystalline core) and in the non-apatitic environments (amorphous surface layer)^45^. We first investigated their exact localisation through the examination of ^31^P-filtered ^1^H ssNMR spectra of hydrated (fresh) *vs* dry 2-year-old sheep bone tissue samples (Fig. S8A). The comparison of the two spectra demonstrates that all the protons from the HPO_4_^2−^ ions undergo fast chemical exchanges with the protons from free water molecules present in the extracellular fluid. Indeed, in the case of the fresh bone tissue sample, the structural water resonance at δ(^1^H) = 5.2 ppm together with the broad signal in the range of δ(^1^H) = 7–17 ppm now safely attributed to HPO_4_^−^ ions, are no longer detected. Instead, a sharp and intense signal of strongly bound water molecules is now observable at δ(^1^H) = 4.8 ppm. A similar behaviour was observed for the biomimetic Carbonated HydroxyApatite (CHA-SBF) sample that was studied in dry conditions and soaked in water (Fig. S8B). These observations make clear that all the HPO_4_^2−^ ions are easily accessible to H_2_O molecules and, therefore, are located near the surface of the particles within the so-called amorphous surface layer that coats bone mineral particles^11,16^.

Second, we have also investigated whether or not HPO_4_^2−^ ions are present in the hydroxyapatite’s crystal lattice of bone mineral particles. To this end, we studied a synthetic HPO_4_^2−^-substituted HA sample. This sample, named HPO_4_^2−^substituted HA, does not possess non-apatitic environments in the form of an amorphous surface layer so that all its HPO_4_^2−^ ions are allegedly localized within the hydroxyapatite’s crystal lattice. The 2D {^1^H}^31^P HetCor ssNMR spectrum of HPO_4_^2−^-substituted HA reveals that the ^31^P NMR chemical shift of its HPO_4_^2−^ ions is quite different than in the case of bone mineral: δ(^31^P) = 1.5 ppm *vs* δ(^31^P) = 3.2 ppm, respectively (Fig. S9). Further, the presence of HPO_4_^2−^ ions in the hydroxyapatite’s crystal lattice of HPO_4_^2−^substituted HA was confirmed with the help of an ^1^H homonuclear dipolar coupling-based ssNMR experiment: the 2D ^1^H–^1^H Single Quantum–Double Quantum (SQ–DQ) correlation experiment (Fig. S10A) which can reveal short ^1^H-^1^H spatial proximities (a few Å). A cross-peak on the left side of the diagonal is observable. It spatially correlates the HPO_4_^2−^ ions region in the range of δ(^1^H) = 7–12 ppm with the OH^−^ ions region at around δ(^1^H) = 0.0 ppm. Such off-diagonal signal demonstrates the incorporation of HPO_4_^2−^ ions within the hydroxyapatite’s crystal lattice of HPO_4_^2−^-substituted HA. In particular, a spatial correlation of those HPO_4_^2−^ ions with a resonance at around δ(^1^H) = 1.0 ppm is highlighted (see red dotted lines); it corresponds to OH^−^ ions close to HPO_4_^2−^ ions that were also observed in the ^1^H single pulse (SP) MAS ssNMR spectrum, see Fig. S9A. Importantly, the same experiment recorded on the biomimetic Carbonated HydroxyApatite (CHA-SBF) sample does not exhibit such spatial proximity between OH^−^ and HPO_4_^2−^ ions (Fig. S10B). These observations not only demonstrate that the HPO_4_^2−^ ions that compose CHA-SBF are concentrated within the so-called amorphous surface layer, but also suggest that the same scenario applies to bone mineral particles.

**Figure 4:**
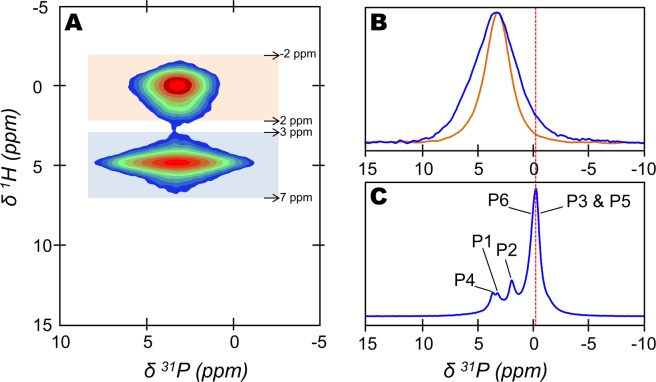
Implication of octacalcium phosphate (OCP) in the non-apatitic environments of bone mineral. (**A**) Two-dimensional (2D) {^1^H}^31^P Heteronuclear Correlation (HetCor) magic angle spinning (MAS) solid-state Nuclear Magnetic Resonance (ssNMR) spectrum of a fresh 2-year-old sheep bone tissue sample (contact time, t_CP_ = 1000 µs). Signal intensity increases from blue to red. (**B**) One-dimensional (1D) individual ^31^P NMR signal of the H_2_O and HPO_4_^2−^-containing non-apatitic environments attributed to the amorphous surface layer that coats bone mineral particles (blue line); and 1D individual ^31^P NMR signal of the OH^−^-containing apatitic environments that compose the internal crystalline core of bone mineral particles (orange line). These individual ^31^P NMR signals were generated from the 2D {^1^H}^31^P HetCor ssNMR spectrum shown in (A). To do so, the F2 slices taken at the bound water molecules position [from δ(^1^H) = 3 to 7 ppm, blue area] and hydroxyl ions position [from δ(^1^H) = −2 to 2 ppm, orange area] in F1 have been summed. (**C**) 1D ^31^P CP MAS ssNMR spectrum (t_CP_ = 1000 µs) of a synthetic octacalcium phosphate (OCP) sample. P1 to P6 correspond to the six different phosphate groups present in the OCP crystal lattice according to the work of Davies *et al*.^60^. The red dashed-line marks the most intense resonance in the signal of OCP which is not detected in bone mineral (**B**).

Lastly, since octacalcium phosphate (OCP) has recently been proposed as a component of bone mineral^20^, the question whether the HPO_4_^2−^ ions detected in the bone mineral of our 2-year-old sheep bone tissue sample originate from OCP environments needs to be raised. In this direction, the 2D {^1^H}^31^P HetCor MAS ssNMR spectrum of a fresh and intact 2-year-old sheep bone tissue sample is shown in Fig. 4A. Again, the upper correlation peak corresponds to the OH^−^ and PO_4_^3−^ containing apatitic environments that compose the internal crystalline core of bone mineral particles. The lower correlation peak corresponds to H_2_O and HPO_4_^2−^-containing non-apatitic environments whose individual ^31^P NMR signal is revealed in Fig. 4B (blue line). This signal is in the form of a single broad resonance whose maximum intensity is at δ(^31^P) = 3.2 ppm, and whose lineshape and linewidth are practically identical to those of the signal of a synthetic sample of amorphous calcium phosphate (ACP) recorded in similar experimental conditions^11^. In contrast, the ^31^P NMR signal of OCP recorded in similar conditions is in the form of three resolved resonances from δ(^31^P) ∼ 3.6 ppm to δ(^31^P) ∼ −0.2 ppm (Fig. 4C). The latter upfield resonance is here the most intense and arises from two overlapping peaks at δ(^31^P) ∼ −0.1 and ∼ −0.3 ppm. They correspond to the two HPO_4_^2−^ groups (P5 and P6) and one of the two PO_4_^3−^ groups (P3) present in the hydrated layer of the OCP crystal lattice^60^. It is worth emphasizing that such upfield signal appears to be absent from the ^31^P NMR signal of the H_2_O and HPO_4_^2−^-containing non-apatitic environments of bone mineral (Fig. 4B). Further, based on a customized ssNMR experiment that was used to probe the long-range spatial proximities among the hydrogen-bearing species in bone mineral, we previously demonstrated that the H_2_O and HPO_4_^2−^-containing non-apatitic environments and the OH^−^-containing apatitic environments belong to the same particle^11^. According to these results that were obtained from intact bone tissue samples, we demonstrated that the H_2_O and HPO_4_^2−^-containing non-apatitic environments evidenced here do not originate from OCP but can be safely attributed to the amorphous surface layer that coats bone mineral particles.

### Quantification of HPO_4_^2−^ ions in bone mineral

**Figure 5:**
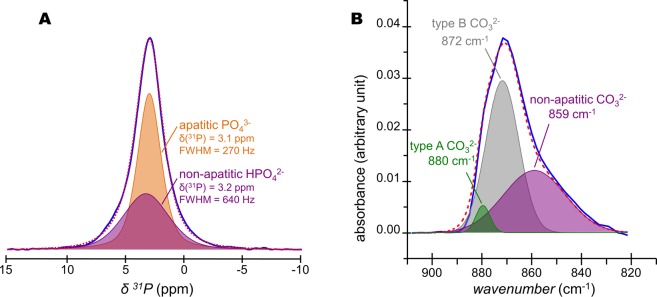
Quantification of HPO_4_^2−^ and CO_3_^2−^ ions present in bone mineral. (**A**) Quantitative ^31^P single pulse (SP) magic angle spinning (MAS) solid-state Nuclear Magnetic Resonance (ssNMR) spectrum of a fresh 2-year-old sheep bone tissue sample (blue line) and its corresponding fitting (red dashed line) with two peaks. Those two peaks, whose lineshape and linewidth were revealed in Fig. 4B, correspond to the PO_4_^3−^-containing internal crystalline core in the form of hydroxyapatite (orange peak) and the HPO_4_^2−^-containing non-apatitic environments in the form of an amorphous surface layer (purple peak). (**B**) Fourier Transform-Infrared (FT-IR) spectrum of the ν_2_(CO_3_) vibration mode for a 2-year-old sheep bone tissue sample (blue line) and its corresponding fitting (red dashed line). Type B CO_3_^2−^ ions occupy the PO_4_^3−^ sites within the hydroxyapatite’s crystal lattice; type A CO_3_^2−^ ions occupy the OH^−^ sites within the hydroxyapatite’s crystal lattice; whereas non-apatitic CO_3_^2−^ are present within the amorphous surface layer that coats bone mineral particles.

The quantification of the HPO_4_^2−^ ions present in bone mineral were undertaken. To this end, the lineshape and linewidth of the individual ^31^P NMR signals of the OH^−^ and PO_4_^3−^-containing internal crystalline core [δ(^1^H) = 3.1 ppm, full width at half maximum (FWHM) = 270 Hz] and the H_2_O and HPO_4_^2−^-containing non-apatitic environments (amorphous surface layer) [δ(^1^H) = 3.2 ppm, FWHM = 640 Hz] that were revealed in Fig. 4B, were used in the fitting of the quantitative ^31^P single pulse (SP) MAS ssNMR spectrum of a fresh 2-year-old sheep bone tissue sample (Fig. 5A). The molar percentage proportion of HPO_4_^2−^ and PO_4_^3−^ ions in bone mineral were found to be about 50/50 ± 5%. As suggested by our observations in Figs S9 and S10, this calculation was made assuming that the molar proportion of HPO_4_^2−^ ions in the internal crystalline core of the particles was close to 0%. Such a high molar proportion of HPO_4_^2−^ ions present in the amorphous surface layer reflects the small size of bone mineral particles: ∼1–5 nm in thickness, ∼10–40 nm in width, and ∼20–100 nm in length^1,61–63^. Since we showed that the HPO_4_^2−^ ions are concentrated within the amorphous surface layer, we can now estimate the average thickness of this layer for a 2-year-old sheep bone tissue sample. Here we consider a nanosized platelet with a thickness of 4.0 nm, and we assume that the densities of phosphate atoms present in the hydroxyapatite’s crystal lattice and in the amorphous surface layer are equivalent. In such scenario, the thickness of the internal crystalline core is about 2.4 nm (i.e., which is about twice the size of the hexagonal unit cell of hydroxyapatite^64^ along the crystallographic axes a and b; a = b = 0.94 nm); whilst the thickness of the outer amorphous surface layer can be estimated to be about 0.8 nm (i.e., which then corresponds to the size of the hexagonal unit cell of hydroxyapatite along a and b, and, hence, is equivalent to the stacking of only two phosphate ions). One should be conscious that these are average values that correspond to the sum of the contributions of all the inorganic phosphate ions present in our 2-year-old sheep bone tissue sample. These results might be different for older specimen in which the proportion of the non-apatitic environments might be less^65^ due to bone mineral maturation: the progressive transformation of the amorphous surface layer into apatitic environments^15^. Nevertheless, the thickness of the surface layer determined here (0.8 nm) is in good agreement with the estimated sizes proposed in some previous studies: about the size of one phosphate unit in fluorapatite-gelatine mesocrystals^66^; and about 1–2 nm in synthetic hydroxyapatites^32,67,68^.

### Update on bone mineral chemical composition

The results presented here and elsewhere^45^ suggest that the average chemical composition of mature cortical bone mineral proposed by Legros *et al*.^27^, Ca_8.3□1,7_(PO_4_)_4.3_(HPO_4_ or CO_3_)_1.7_(OH or ½ CO_3_)_0,3□1.7_, must be reconsidered. Indeed, this formula does not only disregard the presence of the amorphous surface layer whose chemical composition greatly varies with respect to the apatitic environments present in the internal crystalline core of the particles, but also underestimates the molar proportion of HPO_4_^2−^ ions. To propose an up to date formula of our 2-year-old sheep bone tissue sample, we needed firstly to determine the weight proportion of CO_3_^2−^ ions. A value of 4.8% with a major contribution in B-type carbonates was found through FT-IR analyses^49^ (Fig. 5B), which is in accordance with the values found for other bone mineral samples^3^. In addition, the following parameters were considered: *(i)* the particles must remain electrically neutral (both the internal crystalline core and the amorphous surface layer); *(ii)* the molar proportion of HPO_4_^2−^ ions relative to the overall amount of inorganic phosphate ions is constrained close to 50% according to the present study; *(iii)* the degree of carbonation should be close to the experimental value (4.8% w/w); *(iv)* the overall Ca/(P + C) molar ratio should remain acceptable for a bone tissue sample, i.e. in the range of 1.2–1.5^3^ and, last, *(v)* the proportion of A-type, B-type, and non-apatitic carbonate ions present in the amorphous surface layer should remain in accordance with the FT-IR data. Once all of these constraints have been amalgamated, the average chemical composition of the mature cortical bone mineral from our 2-year-old sheep bone tissue sample can be approximated as follows: Ca_7.5_(PO_4_)_2.8_(HPO_4_)_2.6_(CO_3_)_0.6_(OH)_0,2_. One should note that this average chemical composition solely corresponds to our specific bone tissue sample according to our own experimental results. Hence this formula is not universal since variability occurs among bone specimens depending, in particular, on the specie, the age, the food supply and their degree of maturation.

## Conclusions

In this work, we determined the H•••P distances (2.1–2.3 Å) within the acidic phosphate ions that compose bone mineral, and, hence, shown that they undeniably correspond to POH groups in inorganic HPO_4_^2−^ ions. The presence of HPO_4_^2−^ ions in bone mineral was therefore demonstrated based on accurate interatomic distance measurements. Further, in contrast to what was previously proposed, our results suggest that these HPO_4_^2−^ ions are concentrated at the surface of bone mineral particles within the so-called amorphous surface layer since they were not detected within the internal crystalline core in the form of hydroxyapatite in our bone mineral proxy sample. Our results also indicate that the amount of HPO_4_^2−^ ions present in bone mineral is higher than previously determined in previous studies. Indeed, our calculations show that at least half of the overall amount of inorganic phosphate ions that compose bone mineral are in the form of monohydrogen-phosphate ions. As a result, the average chemical composition of the mature cortical bone mineral from our 2-year-old sheep bone tissue sample was approximated as follows: Ca_7.5_(PO_4_)_2.8_(HPO_4_)_2.6_(CO_3_)_0.6_(OH)_0,2_. According to the similarities between sheep and humans, not only in terms of bone and joint structure, but also in terms of bone regeneration and metabolism^69^, this methodological and analytical approach may be translatable to human bones for which a comparable quantification attempt has been undertaken^70^.

**Figure 6:**
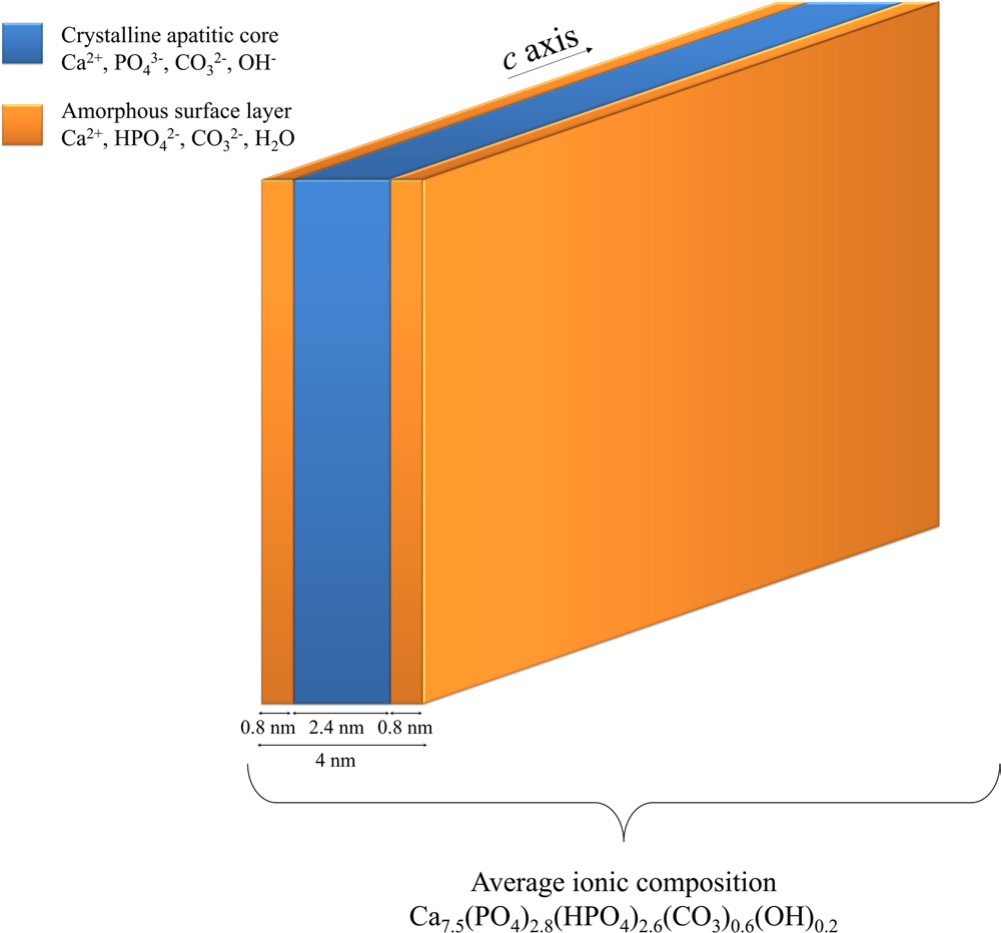
Chemical and structural model of mature bone mineral particles. Schematic representation of platelet-shaped mature bone mineral particles including dimensions and ionic composition according to the results obtained from our 2-year-old sheep bone tissue sample. They are composed by an internal crystalline core in the form of carbonated hydroxyapatite coated by an amorphous layer in which the HPO_4_^2−^ ions are concentrated.

In summary, the present study provides unprecedented insights into the chemical composition and structural features of bone mineral at the atomic scale; and, hence, embodies a key step to design an accurate chemical and structural model of mature bone mineral particles in their biological environments (Fig. 6). Such model is of primary importance to predict bone mineral chemistry and reactivity *in vivo* with an overarching objective of enhancing our understanding of processes involved in healthy and pathological bone formations. In this direction, our results emphasize that the surface chemistry and reactivity of bone mineral are driven by metastable amorphous environments rich in monohydrogen-phosphate ions, rather than by stable crystalline environments with hydroxyapatite structure. As such, the recognition mechanisms at the biomineral-biomolecule (collagen, non-collagenous proteins, etc.) interface, which are long-standing questions in the field of bone biomineralization^71,72^ for shedding light on nucleation and growth processes, must be reconsidered. Further, our results also show the importance of bone mineral surface chemistry in the control of the homeostasis of phosphate ions (i.e., the second ionic buffer in the human body fluids along with carbonates). Last, the analytical tools reported here could be very advantageous for the study of other mineralized tissues in various organisms, including corals^73^ and bivalve mollusks^74^ in which the presence of interfacial monohydrogen-carbonate ions localized in highly-disordered environments have been proposed.

## Supplementary information

Supporting Information
