# Supplementary material for: Bone mineral: new insights into its chemical composition
Source: Sci Rep. 2019 Jun 11;9:8456. doi: 10.1038/s41598-019-44620-6 (PMC6560110; doi:10.1038/s41598-019-44620-6)
Supplement: Supplementary file 1 — Supporting Information [file 41598_2019_44620_MOESM1_ESM.docx]

Supporting information for

**Bone mineral: new insights into its chemical composition**

Stanislas Von Euw,^a,b‡^ Yan Wang,^a‡^ Guillaume Laurent,^a^ Christophe Drouet,^c^ Florence Babonneau,^a^ Nadine Nassif,^a^ Thierry Azaïs^a*^

*^a^ Sorbonne Université, CNRS, Collège de France, Laboratoire de Chimie de la Matière Condensée de Paris (LCMCP), 4, place Jussieu, F-75005, Paris, France.*

*^b^Present address: Trinity Centre for Bioengineering, Trinity Biomedical Sciences Institute, Trinity College Dublin (TCD), Dublin 2, Ireland.*

*^c^ CIRIMAT, UMR CNRS/INP/UPS 5085, Université de Toulouse, Ensiacet, 4 allée Emile Monso, F-31030, Toulouse, France*

^‡^ These two authors contributed equally to this work

*Corresponding Authors: thierry.azais@upmc.fr

**This PDF file includes:**

Figures S1 to S10

References

Figure S1

**Fig. S1**. Schematic description of the {^1^H-^31^P}^1^H double cross polarization (CP) magic angle spinning (MAS) solid-state Nuclear Magnetic Resonance (ssNMR) experiment. This experiment consists of a double CP transfer conducted in a “there-and-back” manner (^1^H → ^31^P → ^1^H) to detect the ^1^H nucleus which is the high-γ nucleus (inverse detection). Two CP transfers are executed: a first transfer from ^1^H to ^31^P (characterized by contact time 1, t_CP_1) is followed by a transfer back from ^31^P to ^1^H (characterized by contact time 2, t_CP_2), and are separated by ^1^H saturation pulses in order to remove the residual ^1^H Zeeman signal.

Figure S2

**Fig. S2.** Hartmann-Hahn profile recorded from the biominetic Carbonated HydroxyApatite sample that was precipitated directly from a modified simulated body fluid solution (CHA-SBF). The radio frequency (RF) ν_RF_(^1^H) was fixed, whereas ν_RF_(^31^P) was varied. Maxima in the Hartmann-Hahn profile verify the following condition: ν_RF_(^31^P) = ν_RF_(^1^H) ± nν_MAS_ (ν_MAS_ = 14 kHz; and n = 1, 2).

Figure S3

**Fig. S3**. {^1^H-^31^P}^1^H double cross polarization (CP) magic angle spinning (MAS) solid-state Nuclear Magnetic Resonance (ssNMR) spectrum of a dry 2-year-old sheep bone tissue sample (contact times, t_CP_1 = t_CP_2 = 15000 µs) in the OH^-^ resonance region (blue line); and its corresponding fitting (red dashed line) with three peaks at δ(^1^H) = -0.7 (purple); 0.0 (grey); and 0.9 (blue) ppm. The origin of such complex line shape is not trivial. This could arise from the presence of various ionic substitutions (Na^+^, CO_3_^2-^, HPO_4_^2-^, Mg^2+^…) and vacancies in bone hydroxyapatite that induce a modification of the ^1^H NMR chemical shift of hydroxyl ions at the vicinity of substituted ions. For instance, it has been shown for synthetic carbonated hydroxyapatite that OH^-^ ions close to CO_3_^2-^ ions in B-type substitution exhibit a ^1^H NMR signal shifted to 1.0 ppm^1,2^. Nevertheless, the signal arising from OH^-^ in bone hydroxyapatite is significantly broader than for most of the synthetic hydroxyapatite samples. In addition, the confinement induced by the collagen network during mineral growth and maturation might lead to smaller and less crystallized particles compared to synthetic hydroxyapatite particles^3^.

Figure S4

**Fig. S4.** {^1^H-^31^P}^1^H double cross polarization (CP) magic angle spinning (MAS) solid-state Nuclear Magnetic Resonance (ssNMR) spectrum of a dry 2-year-old sheep bone tissue sample (blue lines) and their corresponding fitting results (red dashed line). Contact time 1 (t_CP_1) was 1000 µs for all spectra, while contact time 2 (t_CP_2) was varied from 75 µs to 1000 µs. Shown are the best fitting results using one single peak (left column) or two peaks (right column) for the broad signal attributed to acidic phosphate species observable in the range of δ(^1^H) = 7-17 ppm. In addition, the residual structural water resonance was fitted with a single peak centered at δ(^1^H) = 5.2 ppm, while the complex hydroxyl resonance centered at δ(^1^H) = 0.0 ppm was fitted according to the fitting results shown in Fig. S3. Positions and linewidths of the different peaks were kept constant for each simulation.

Figure S5

**Fig. S5**. (**A**) {^1^H-^31^P}^1^H double cross polarization (CP) magic angle spinning (MAS) solid-state Nuclear Magnetic Resonance (ssNMR) spectrum of a synthetic monetite (CaHPO_4_) sample. Contact time 1 (t_CP_1) was 1000 µs for all spectra, while contact time 2 (t_CP_2) was varied from 75 µs to 1000 µs. Black dashed lines are guidelines for the eyes. (**B**) Numerical modelling according to Equation (1) of the magnetization evolution of the upfield resonance at δ(^1^H) = 13.0 ppm attributed to HPO_4_^2-^ ions; together with the calculations of their respective dipolar constants (D_PH_) and P•••H distances (d_PH_).

Figure S6

**Fig. S6.** (**A**) {^1^H-^31^P}^1^H double cross polarization (CP) magic angle spinning (MAS) solid-state Nuclear Magnetic Resonance (ssNMR) spectrum of a synthetic monetite (CaHPO_4_) sample (blue line); and its corresponding fitting (red dashed line) with two peaks respectively centered at δ(^1^H) = 13.0 (grey) and 15.8 (purple) ppm both attributed to HPO_4_^2-^ ions. Contact time 1 (t_CP_1) was 2000 µs, while contact time 2 (t_CP_2) was 300 µs. (**B**) Numerical modelling according to Equation (1) of the magnetization evolution of the downfield resonance at δ(^1^H) = 15.8 ppm, which is more accurate than the modelling of the magnetization evolution of the upfield δ(^1^H) = 13.0 ppm resonance shown in Fig. S5B. Dipolar oscillations are observed, but the corresponding H•••P distance is a little bit shorter than expected (D_PH_ = 6863 Hz; and d_PH_ = 1.92 Å - Table 1). This could be due to the fact that the protons from the δ(^1^H) = 15.8 ppm resonance were found to be shared between two HPO_4_^2-^ groups by neutron diffraction analysis^4^. As a consequence, our numerical modeling within the single spin pair approximation might be not adequate in this particular scenario.

Figure S7

**Fig. S7.** (**A**) {^1^H-^31^P}^1^H double cross polarization (CP) magic angle spinning (MAS) solid-state Nuclear Magnetic Resonance (ssNMR) spectrum of the biomimetic Carbonated HydroxyApatite (CHA-SBF) sample. Contact time 1 (t_CP_1) was 1000 µs for all spectra, while contact time 2 (t_CP_2) was varied from 75 µs to 1250 µs. Black dashed lines are guidelines for the eyes. (**B**) Numerical modelling according to Equation (1) of the magnetization evolution of the signal in the range of δ(^1^H) = 7-17 ppm attributed to acidic phosphate species; together with the calculations of its respective dipolar constant (D_PH_) and P•••H distance (d_PH_).

Figure S8

**Fig. S8**. (**A**) ^31^P-filtred ^1^H magic angle spinning (MAS) solid-state Nuclear Magnetic Resonance (ssNMR) spectra of dry (top) and fresh (bottom) 2-year-old sheep bone tissue samples. (**B**) ^31^P-filtred ^1^H MAS ssNMR spectra of dry (top) and wet (bottom) biomimetic Carbonated HydroxyApatite (CHA-SBF) samples. The spectra of the dry bone samples were obtained from {^1^H-^31^P}^1^H double cross polarization (CP) experiments with the following contact times: t_CP_1 = t_CP_2 = 1000 µs. The spectra of the hydrated samples (fresh bone tissue and wet CHA-SBF) were obtained from two-dimensional (2D) {^1^H}^31^P Heteronuclear Correlation (HetCor) experiments (contact time, t_CP_ = 1000 µs) from which the ^1^H projections of the F1 dimensions were extracted.

Figure S9

**Fig. S9.** (**A**) Two-dimensional (2D) {^1^H}^31^P Heteronuclear Correlation (HetCor) magic angle spinning (MAS) solid-state Nuclear Magnetic Resonance (ssNMR) spectrum of HPO_4_^2-^-substituted HA (contact time, t_CP_ = 1000 µs). Signal intensity increases from blue to red. (**B**) Sums of the F2 (^31^P) slices taken at the HPO_4_^2-^ ions position [from δ(^1^H) = 7.0 to 7.0 ppm] in the F1 dimensions of the 2D {^1^H}^31^P HetCor spectra of HPO_4_^2-^-substituted HA (top) and a dry 2-year-old sheep bone tissue sample (bottom).

Figure S10

**Fig. S10**. Two-dimensional (2D) ^1^H–^1^H Single Quantum–Double Quantum (SQ–DQ) correlation magic angle spinning (MAS) solid-state Nuclear Magnetic Resonance (ssNMR) spectra of HPO_4_^2-^-substituted HA (**A**) and the biomimetic Carbonated HydroxyApatite (CHA-SBF) sample (**B**). Signal intensity increases from blue to red. The cross-peak between HPO_4_^2-^ and OH^-^ ions from the hydroxyapatite’s crystal lattice of HPO_4_^2-^-substituted HA is highlighted with dashed red lines; the corresponding cross-peak on the right side of the diagonal is absent due to signal truncation and T_1_ noise.

***References:***

1. Babonneau, F., Bonhomme, C., Hayakawa, S. & Osaka, A. Solid State NMR Characterization of Nano-crystalline hydroxy-carbonate Apatite Using 1H-31P-13C Triple Resonance Experiments. *MRS Online Proc. Libr. Arch.* **984**, (2006).

2. de Leeuw, N. H. Computer simulations of structures and properties of the biomaterial hydroxyapatite. *J. Mater. Chem.* **20**, 5376–5389 (2010).

3. Wang, Y. *et al.* Impact of collagen confinement vs. ionic substitutions on the local disorder in bone and biomimetic apatites. *Mater. Horiz.* **1**, 224–231 (2014).

4. Catti, M., Ferraris, G. & Mason, S. A. Low-temperature ordering of hydrogen atoms in CaHPO4 (monetite): X-ray and neutron diffraction study at 145 K. *Acta Crystallogr. B* **36**, 254–259 (1980).
